# Next-generation sequencing of von Willebrand factor and coagulation factor VIII genes: a cross-sectional study in Croatian adult patients diagnosed with von Willebrand disease

**DOI:** 10.3325/cmj.2022.63.166

**Published:** 2022-04

**Authors:** Ivana Lapić, Margareta Radić Antolic, Ana Boban, Désirée Coen Herak, Dunja Rogić, Renata Zadro

**Affiliations:** 1 Department of Laboratory Diagnostics, University Hospital Center Zagreb, Zagreb, Croatia; 2 Division of Hematology, Department of Internal Medicine, Zagreb, Croatia; 3 Faculty of Pharmacy and Biochemistry, University of Zagreb, Zagreb, Croatia; 4 Medical Biochemistry Laboratory, St Catherine Specialty Hospital, Zagreb, Croatia

## Abstract

**Aim:**

To identify the von Willebrand factor (VWF) gene variant status in Croatian adult patients diagnosed with von Willebrand disease (VWD), provide differential diagnosis of VWD subtypes, and identify patients with mild hemophilia A (HA) who were earlier misdiagnosed as VWD.

**Methods:**

Coagulation testing included determination of VWF gain-of-function mutant glycoprotein Ib binding activity (VWF:GPIbM), VWF antigen, VWF collagen-binding activity, and multimeric analysis. Genetic analysis of VWF and FVIII genes was performed with next-generation sequencing (NGS).

**Results:**

The study enrolled 50 patients (72% women; median age 37 years, range 18-75) from 44 unrelated families. Fourteen patients were heterozygous for VWF gene variants compatible with type-1 VWD. Twelve had variants associated with type 2, of whom seven were classified as type 2A, four as type 2B, and one as type 2N. Six type-3 VWD patients were either homozygotes for null variants or combined heterozygotes. Eleven variants within the VWF gene were novel. Three female patients had variants within the FVIII gene, and were re-classified as mild-HA carriers, of whom one had causative novel variants both within VWF and FVIII genes. Fifteen patients remained without a defined genetic cause of their disorder, of whom five had VWF:GPIbM levels below 50%.

**Conclusion:**

Croatian adult patients with VWD have considerable genetic heterogeneity. NGS of both VWF and FVIII genes provided accurate differential diagnosis of VWD subtypes and distinction of VWD from mild HA.

Von Willebrand disease (VWD) is the most frequent autosomally inherited bleeding disorder caused by quantitative deficiency or qualitative changes in the von Willebrand factor (VWF) protein structure. The prevalence of symptomatic cases among the general population ranges from 0.01% to 1%. This large multimeric plasma glycoprotein mediates platelet adhesion and aggregation at the vascular injury site through interaction with the platelet glycoprotein Ib (GPIb) and collagen in the subendothelium. Additionally, it transports and stabilizes coagulation factor VIII (FVIII) in circulation (1-3). Multiple VWF functions in hemostasis are regulated by different regions within the encoding gene, which is located on chromosome 12 and comprises 52 exons. To date, more than 700 unique disease-associated variants have been discovered throughout the whole VWF gene, affecting either VWF assembly, secretion, proteolysis, clearance, and/or binding affinity to GPIb, collagen, and FVIII (1,4). Any impairment of VWF function results in bleeding phenotypes, whose severity depends on the causative pathophysiological mechanism and the remaining levels of functional VWF (2). The majority of patients present with mild to moderate mucocutaneous bleeding and prolonged bleeding after trauma (5). The most severe cases suffer from spontaneous bleeding episodes, including joint, muscle, and intracerebral hemorrhage (5). Up to 80% of all VWD cases have a certain quantitative deficiency of a functionally and structurally normal VWF, which is classified as type-1 VWD. Overall, 15%-20% of cases have qualitative disorders within VWF, classified as subtypes 2A, 2B, 2M, and 2N relative to the underlying structural and functional defect. Specifically, type 2A is characterized by selective loss of high (HMWM) and/or intermediate molecular weight multimers, which results in a decreased ability of VWF to bind GPIb and collagen. Disorder of VWF in type 2B is related to enhanced binding of VWF to GPIb, thus causing increased platelet clearance and loss of HMWM. Type 2M is caused by decreased binding of VWF to GPIb, and is usually associated with a normal multimeric profile, while type 2N results in decreased VWF binding to FVIII in circulation. Type-3 VWD, the most severe form, characterized by undetectably low VWF levels, occurs in approximately 1 in a million people (2,6). Importantly, about 35% of all patients with mildly decreased VWF levels lack distinctive genetic markers. This is considered a separate entity termed “low VWF” (7).

Determination of VWF activity in terms of its capability to bind platelet GPIb, together with VWF antigen (VWF:Ag) levels and FVIII coagulant activity (FVIII:C), represents the mainstay of VWD laboratory diagnostics. VWF activity can be determined either with ristocetin cofactor activity assays that measure the ability of VWF to bind GPIb in the presence of ristocetin, or with immunoturbidimetric assays that use latex microparticles coated with GPIb containing two gain-of-function mutations that enhance VWF binding (VWF:GPIbM). Distinguishing different type-2 VWD subtypes reguires additional investigation of VWF structural and functional features by means of activity assays that measure VWF binding to collagen (VWF:CBA) as well as multimeric analysis (5). Proper VWD classification is the basis for adequate treatment and patient counseling (6). Given the multifunctional nature and structural complexity of VWF, heterogeneity of underlying genetic variants, and variable bleeding tendency, accurate differential diagnosis of VWD is often challenging. The exact nature of causative VWF defect might remain incompletely revealed when commonly available laboratory assays are used (6,8). Patients presenting with ambiguous mild-bleeding phenotypes coupled with borderline levels of VWF (30%-50%) are especially hard to be properly characterized (7). Difficulties may also arise due to similarities in clinical presentation and laboratory results to patients with the mild form of hemophilia A (HA), especially in type 2N VWD characterized by similarly low FVIII levels. HA is a well-known inherited bleeding disorder characterized by decreased FVIII levels (9-12).

Simultaneous genetic analysis of both VWF and FVIII genes with next-generation sequencing (NGS) allows a definitive differential diagnosis of VWD subtypes and possible distinction from mild HA. It also reveals the inheritance risk for family members. This approach enabled unambiguous diagnosis of either VWD or mild HA in patients with mild bleeding disorders (10,11). In addition, NGS of the VWF gene in Spanish patients resulted in reclassification to a different VWD subtype in approximately one quarter of participants (13). Each study conducted so far identified a subset of novel, disease-associated variants, confirming the complexity of the underlying genetic basis of VWD and further clarifying heterogeneous clinical presentation (13-17). The presence of compound heterozygosity in 37.5%-63.3% of patients additionally contributes to disease heterogeneity (14-16). Furthermore, distribution and frequency of variants within the VWF differ relative to geographic localization and ethnicity (13-15,17). Given the lack of such studies in Croatia, the genetic basis of VWD among Croatian population remains unknown.

The aim of the present study was to provide molecular diagnosis by means of NGS in Croatian adult patients diagnosed with VWD, and thereby reveal the VWF gene variant status and associated VWD subtypes. Additionally, by simultaneous analysis of both VWF and FVIII genes we aimed to identify patients with mild HA who were earlier misdiagnosed as VWD.

## Patients and methods

### Study setting and participants

This cross-sectional study enrolled Croatian adult patients previously diagnosed with VWD. Diagnosis was established based on either VWF:GPIbM levels below 50%, clinical presentation indicative of VWD that included mild to moderate bleeding tendency (mucocutaneous bleeding, prolonged bleeding after trivial injuries, excessive bruising, menorrhagia in women) or severe bleeding symptoms (gastrointestinal bleeding, hematuria, joint and muscle bleeds, intracerebral hemorrhage), and/or positive family history of VWD.

**Figure 1:**
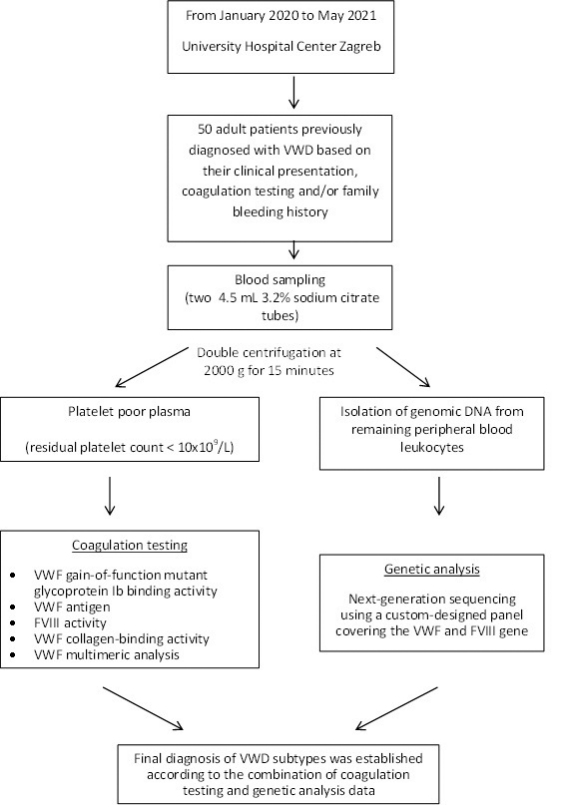
Study protocol. VWF – von Willebrand factor; VWD – von Willebrand disease; FVIII – coagulation factor VIII.

The participants were recruited from January 2020 to May 2021 during their regular medical visits to the Hemophilia Center, Department of Internal Medicine, University Hospital Center Zagreb. Laboratory analyses were performed at the Department of Laboratory Diagnostics, University Hospital Center Zagreb. The study protocol is presented in Figure 1.

The study was approved by the University Hospital Center Zagreb Ethics Committee (8.1-19/293-2, 02/21 AG) and Ethics Committee for Experimentation of the University of Zagreb Faculty of Pharmacy and Biochemistry (251-62-03-20-13). All participants signed an informed consent before enrollment.

### Blood samples

Two 4.5-mL 3.2% sodium citrate tubes (Greiner Bio-One, Kremsmünster, Austria) were drawn from each participant. Platelet poor-plasma needed for coagulation testing with residual platelet count lower than 10 × 10^9^/L was obtained after double sample centrifugation at 2000 g for 15 minutes at room temperature within two hours from blood collection. Plasma samples were aliquoted, stored at -80 °C, and analyzed in batches within the recommended timeframe for each assay. Genomic DNA was extracted from peripheral blood leukocytes with the MagNA Pure Compact instrument (Roche Diagnostics, Basel, Switzerland).

### Coagulation testing

Coagulation testing included determination of VWF:GPIbM, VWF:Ag, FVIII:C, VWF:CBA and multimers analysis. Both VWF:GPIbM and VWF:Ag were determined with commercially available automated immunoturbidimetric assays, while FVIII:C was measured with a one-stage clotting method, on Atellica COAG 360 analyzer (Siemens Healthcare, Marburg, Germany) following original manufacturer's protocols. VWF:CBA reflecting the ability of binding collagen type 3 was determined by using the enzyme-linked immunosorbent assay Technozym VWF:CBA (Technoclone, Vienna, Austria). VWF multimers were analyzed with agarose gel electrophoresis with direct immunofixation with the Hydragel 5 von Willebrand multimers kit on Hydrasys 2 Scan instrument (Sebia, Lisses, France).

### Genetic analysis

NGS was performed with a dedicated, custom-designed panel provided by Integrated DNA Technologies (IDT, Commercial Park Coralville, IA, USA). It comprised all 52 exons, intronic flanking regions, and the promoter of the VWF gene, as well as all 26 exons and the promoter region of the FVIII gene. Libraries were prepared by using the Illumina DNA Prep with Enrichment kit (Illumina, San Diego, CA, USA) according to manufacturer's instructions (18). Specifically, based on the DNA concentrations measured with the Qubit 3 fluorometer (Thermo Fisher Scientific, Waltham, MA, USA), each DNA sample was diluted to an input quantity of 300 mg. Library preparation included tagmentation of genomic DNA by using enrichment bead-linked transposomes, amplification, and indexing, followed by double-sided bead purification. The mean fragment size was expected to be between 300 and 400 bp and was assessed with the DNA 1000 kit on the automated electrophoresis fragment analyzer 4150 TapeStation (both by Agilent Technologies, Santa Clara, CA, USA). The fragmented libraries were then pooled by volume, hybridized with the enrichment probe panel, and amplified. After purification of enrichment libraries with paramagnetic beads, fragment size and molarity were determined with the High Sensitivity DNA kit (Agilent Technologies) on 4150 TapeStation. Each library was diluted to a starting concentration of 4 nM to prepare a multiplexed library pool for sequencing, which was denaturated by adding 0.2 M NaOH, diluted to 20 pM by using the appropriate hybridization buffer, and further diluted to the final loading concentration of 12 pM.

NGS was performed on the Miseq platform (Illumina) by using the 300-cycle Miseq reagent kit v2 with 150-bp paired-end reads. The generated FASTQ files for each patient were imported to the BaseSpace Variant Interpreter (Illumina), where allignment to the hg19 reference genome and variant annotation were carried out. The identified variants were searched in the VWF (4) and FVIII variants databases (9), as well as in the literature. The pathogenicity of novel variants was examined with the VarSome platform (19). Variants classified as “pathogenic” and “likely pathogenic” were considered disease-associated. Based on the results of coagulation testing and genetic analysis, patients were classified as having VWD type 1, type 2A, type 2B, type 2M, type 2N, type 3, or “low VWF.”

### Statistical analysis

Data distribution normality was assessed with the Shapiro-Wilk test. The results are presented as medians and interquartile ranges (IQR). Data were analyzed with the MedCalc statistical software, version 19.5.2 (MedCalc, Ostend, Belgium).

## Results

The study enrolled 50 Croatian adult patients (72% women; median age 37, range 18-75 years) from 44 unrelated families (Table 1). Among them, 32 were identified with disease-associated variants within the VWF gene, of whom 14 were classified as type 1-VWD (Table 2), 12 as type-2 VWD, and 6 as type-3 VWD. In total, 31 different disease-associated variants were found within the VWF gene, comprising 21 missense, 6 stop, and 2 frameshift variants within exons, and two causative variants within intronic splicing sites. Patients with type-1 VWD harbored 13 genetic variants; patients with type-2 VWD harbored 13 genetic variants; and patients with type-3 VWD harbored 6 genetic variants. The homozygous variant c.4975C>T (p.Arg1659Ter) found in the latter group was identified also in one case with type-1 VWD but in a heterozygous state.

**Table 1:** Demographic data and results of coagulation testing according to subgroups of patients with VWD. Results are presented as medians and range if not otherwise indicated*

	Type-1 VWD (N = 14)	Type-2 VWD (N = 12)	Type-3 VWD (N = 6)	Low VWF (N = 5)	Patients without disease-associated variants and normal coagulation testing (N = 10)	Carriers of mild HA (N = 3)
Age, years	40 (20-75)	37 (23-69)	44 (26-54)	22 (18-58)	37 (18-54)	26 (22-35)
Women, n (%)	12 (86)	10 (83)	3 (50)	1 (20)	7 (70)	3 (100)
VWF:GPIbM (%)	44.7 (15.6-83.7)	13.4 (<4.0-126.4)	<4.0-8.3^†^	41.4 (6.9-49.6)	76.2 (57.5-142.4)	52.2 (50.9-56.6)
VWF:Ag (%)	58.6 (18.0-92.3)	51.3 (13.5-102.5)	<4.0-7.3^†^	39.1 (14.7-57.3)	82.2 (65.0-118.8)	53.0 (52.9-62.6)
FVIII:C (%)	72 (22-180)	37 (10-92)	5 (0.8-35)	51 (14-102)	83 (70-220)	23 (19-45)
VWF:CBA (%)	38.6 (14.0-64.7)	23.1 (3.7-79.3)	<1.0-3.5^†^	38.0 (6.1-51.7)	64.5 (52.1-170.0)	38.0 (35.1-46.0)

*Abbreviations: VWD – von Willebrand disease; VWF – von Willebrand factor; HA – hemophilia A; GPIbM – gain-of-function mutant glycoprotein Ib binding activity; Ag – antigen; FVIII:C – coagulation factor VIII; CBA – collagen binding activity

†Due to values below the lower measuring limit for some patients, statistical analysis could not be performed and only the range of obtained results is reported.

**Table 2:** Coagulation testing and genetic analysis of patients with confirmed disease-associated variants within the VWF gene who were classified as type-1 von Willebrand disease*

Cases	Coagulation testing						Genetic analysis				
	VWF:GPIbM (%)	VWF:Ag (%)	VWF:GPIbM/VWF:Ag ratio	FVIII:C (%)	VWF:CBA (%)	Multimers distribution	Gene region	Nucleotide change	Aminoacid change	VWF domain	Variant type

Reference interval	50.0-187.0	50.0-160.0	N/A	50-149	40.0-250.0	N/A					
VWD1-1	15.6	19.5	0.80	72	14.0	Normal	Exon 13	c.1496A>C^‡^	p.Gln499Pro	D2	Missense
VWD1-2	60.8	62.8	0.97	71	41.9	Normal	Exon 13	c.1531A>C^‡^	p.Lys511Gln	D2	Missense
VWD1-3	41.3	67.8	0.61	72	50.5	Normal	Exon 17	c.2269_2270delCT^‡^	p.Leu757ValfsTer22	D2	Frameshift indels
VWD1-4	46.0	60.6	0.76	45	35.3	Normal	Exon 22	c.2878C>T	p.Arg960Trp	D3	Missense
VWD1-5	17.2	18.0	0.96	22	14.2	Normal	Exon 27	c.3614G>A	p.Arg1205His	D3	Missense
VWD1-6	53.3	56.5	0.94	70	46.6	Normal	Exon 28	c.4696C>T	p.Arg1566Ter	A2	Stop gained
VWD1-7	83.7	92.3	0.91	32	64.7	Normal	Exon 28	c.4751A>G	p.Tyr1584Cys	A2	Missense
VWD1-8	43.4	55.4	0.78	92	42.0	Normal	Exon 28	c.4975C>T	p.Arg1659Ter	A2	Stop gained
VWD1-9	58.8	78.1	0.75	89	62.9	Normal	Exon 37	c.6479A>G†	p.Tyr2160Cys	D4	Missense
VWD1-10^†^	36.6	48.5	0.75	104	32.4	Normal	Exon 37	c.6504C>A^‡^	p.Cys2168Ter	D4	Stop gained
VWD1-11^†^	50.0	77.7	0.64	180	32.3	Normal					
VWD1-12	28.7	27.2	1.06	43	18.0	Normal	Exon 37	c.6596G>A^‡^	p.Cys2199Tyr	D4	Missense
VWD1-13	39.7	51.5	0.77	74	28.1	Normal	Intron 37	c.6599-2A>G^‡^	-	-	Splicing
VWD1-14	65.8	65.3	1.01	81	45.3	Normal	Intron 45	c.7729 + 7C>T	-	-	Splicing

*Abbreviations: VWF – von Willebrand factor; VWD – von Willebrand disease; GPIbM – gain-of-function mutant glycoprotein Ib binding activity; Ag – antigen; FVIII:C – coagulation factor VIII; CBA – collagen binding activity; N/A – not applicable.

†Patients are from the same family.

‡Novel disease-associated variants identified within the VWF gene.

Importantly, this study identified 11 novel variants within the VWF gene, of which 7 were associated with type-1 VWD, 1 with type-2A VWD, and 3 with type-3 VWD, all being part of combined heterozygous genotypes.

Fifteen patients were not identified with disease-associated variants, of whom 5 were classified as “low VWF” based on VWF:GPIbM levels below 50%. Two female patients were identified with disease-associated variants within the FVIII gene only, while 1 patient had a causative variant within both the VWF and FVIII gene. These patients were re-classified as carriers of mild HA. Two variants within the FVIII gene were novel.

Of 12 patients with type-2 VWD, 7 were assigned as type 2A, 4 as type 2B, and 1 as type 2N (Table 3).

**Table 3:** Coagulation testing and genetic analysis of patients with confirmed disease-associated variants within the VWF gene who were classified as type-2 von Willebrand disease*

Cases	Coagulation testing						Genetic analysis					VWD subtype
	VWF:GPIbM (%)	VWF:Ag (%)	VWF:GPIbM/VWF:Ag ratio	FVIII:C (%)	VWF:CBA (%)	Multimers distribution	Exon	Nucleotide change	Amino acid change	VWF domain	Variant type	
Reference interval	50.0-187.0	50.0-160.0	N/A	50-149	40.0-250.0	N/A						
VWD2-1	57.9	53.6	1.08	66	58.1	Normal	17	c.2278C>T	p.Arg760Cys	D2	Missense	2N
							20	c.2561G>A	p.Arg854Gln	D'	Missense	
VWD2-2	126.4	102.5	1.23	92	79.3	Normal	28	c.3797C>T	p.Pro1266Leu	A1	Missense	2B
							28	c.3835G>A	p.Val1279Ile	A1	Missense	
VWD2-3^†^	10.9	17.2	0.63	21	10.1	↓HMWM, ↓IMWM	28	c.3829G>T^§^	p.Asp1277Tyr	A1	Missense	2A
VWD2-4^†^	15.3	30.3	0.50	41	22.4	↓HMWM, ↓IMWM						
VWD2-5^‡^	21.8	63.6	0.34	32	34.4	Loss of HMWM	28	c.3946G>A	p.Val1316Met	A1	Missense	2B
VWD2-6^‡^	16.0	54.6	0.29	30	27.8	Loss of HMWM						
VWD2-7	39.8	66.8	0.60	48	42.5	↓HMWM	28	c.4010C>T	p.Pro1337Leu	A1	Missense	2B
VWD2-8	10.7	32.1	0.33	61	23.7	Normal	28	c.4027A>G	p.Ile1343Val	A1	Missense	2A
							28	c.4133C>T	p.Ser1378Phe	A1	Missense	
VWD2-9	7.7	41.9	0.18	24	15.8	↓HMWM, ↓IMWM	28	c.4121G>A	p.Arg1374His	A1	Missense	2A
VWD2-10	<4.0	13.5	N/A	10	3.7	↓HMWM, ↓IMWM	28	c.4517C>T	p.Ser1506Leu	A2	Missense	2A
VWD2-11	5.6	51.6	0.11	39	13.3	Loss of HMWM, ↓IMWM	28	c.4645G>A	p.Glu1549Lys	A2	Missense	2A
VWD2-12	11.4	51.0	0.22	34	20.9	Loss of HMWM, ↓IMWM	28	c.4825G>A	p.Gly1609Arg	A2	Missense	2A

*Abbreviations: VWF – von Willebrand factor; VWD – von Willebrand disease; GPIbM – gain-of-function mutant glycoprotein Ib binding activity; Ag – antigen; FVIII:C – coagulation factor VIII; CBA – collagen binding activity; HMWM – high molecular weight multimers; IMWM – intermediate molecular weight multimers; N/A – not applicable.

†Patients are from the same family.

‡Patients are from the same family.

§Novel disease-associated variant identified within the VWF gene.

Six patients with type-3 VWD had markedly decreased or values below the lower measuring limit for coagulation tests, and were either homozygotes or combined heterozygotes for variants in the VWF gene (Table 4).

**Table 4:** Coagulation testing and genetic analysis of patients with confirmed disease-associated variants within the VWF gene who were classified as type-3 von Willebrand disease*

Cases	Coagulation testing				Genetic analysis					
	VWF:GPIbM (%)	VWF:Ag (%)	FVIII:C (%)	VWF:CBA (%)	Exon	Nucleotide change	Amino acid change	VWF domain	Variant type	Genotype
Reference interval	50.0-187.0	50.0-160.0	50-149	40.0-250.0						
VWD3-1^†^	7.7	7.3	35	3.5	18	c.2435delC	p.Pro812ArgfsTer31	D'	Frameshift Indels	Homozygous
VWD3-2^†^	<4.0	<4.0	4	<1.0						
VWD3-3	<4.0	<4.0	7	<1.0	28	c.4975C>T	p.Arg1659Ter	A2	Stop gained	Homozygous
VWD3-4‡	8.3	<4.0	5	<1.0	4 36	c.319C>T^§^ c.6151A>T^§^	p.Gln107Ter p.Arg2051Ter	D1 D4	Stop gained Stop gained	Heterozygous Heterozygous
VWD3-5‡	<4.0	<4.0	0.8	<1.0						
VWD3-6	<4.0	<4.0	5	<1.0	6 45	c.571T>C^§^ c.7603C>T	p.Trp191Arg p.Arg2535Ter	D1 C4	Missense Stop gained	Heterozygous Heterozygous

*Abbreviations: VWF – von Willebrand factor; VWD – von Willebrand disease; GPIbM – gain-of-function mutant glycoprotein Ib binding activity; Ag – antigen; FVIII:C – coagulation factor VIII; CBA – collagen binding activity; N/A – not applicable.

†Patients VWD3-1 and VWD3-2 are not related.

‡Patients VWD3-4 and VWD3-5 are from the same family.

§Novel disease-associated variants identified within the VWF gene.

Of 5 patients classified as “low VWF,” 1 had remarkably decreased levels of VWF:GPIbM, VWF:Ag and FVIII:C (Table 5). Ten remaining patients without disease-associated variants had results of all phenotypic laboratory assays within the reference intervals, as well as normal distribution of VWF multimers. Three patients had disease-associated variants within the FVIII gene (Table 6).

**Table 5:** Coagulation testing for patients with decreased VWF levels and no identified disease-associated variant within the VWF gene*

Cases	VWF:GPIbM (%)	VWF:Ag (%)	FVIII:C (%)	VWF:CBA (%)	Multimers distribution
Reference interval	50.0-187.0	50.0-160.0	50-149	40.0-250.0	N/A
1	6.9	14.7	14	6.1	Normal
2	30.6	39.1	102	38.0	Normal
3	41.4	33.1	51	27.8	Normal
4	45.5	57.3	98	51.7	Normal
5	49.6	56.3	43	38.0	Normal

*Abbreviations: VWF:GPIbM – von Willebrand factor gain-of-function mutant glycoprotein Ib binding activity; VWF:Ag – von Willebrand factor antigen; FVIII:C – coagulation factor VIII; VWF:CBA – von Willebrand factor collagen binding activity; N/A – not applicable.

**Table 6:** Coagulation testing and genetic analysis of patients with confirmed disease-associated variants within the FVIII gene who were re-classified as carriers of mild hemophilia A*

Cases	Coagulation testing					Genetic analysis					
	VWF:GPIbM (%)	VWF:Ag (%)	FVIII:C (%)	VWF:CBA (%)	Multimers distribution	Gene	Exon	Nucleotide change	Amino acid change	Variant type	Genotype
Reference interval	50.0-187.0	50.0-160.0	50-149	40.0-250.0	N/A						
HA-1	52.2	52.9	45	46.0	Normal	FVIII	21	c.6253G>A^†^	p.Glu2085Lys	Missense	Heterozygous
HA-2	56.6	62.6	23	35.1	Normal	FVIII	4	c.599A>G	p.Glu200Gly	Missense	Heterozygous
HA-3	50.9	53.0	19	38.0	Slightly decreased HMWM and IMWM	FVIII	11	c.1553A>C^†^	p.Lys518Thr	Missense	Heterozygous
						VWF	37	c.6479A>G^†^	p.Tyr2160Cys	Missense	Heterozygous

*Abbreviations: HA – hemophilia A; VWF – von Willebrand factor; GPIbM – gain-of-function mutant glycoprotein Ib binding activity; Ag – antigen; FVIII:C – coagulation factor VIII; CBA – collagen binding activity; HMWM – high molecular weight multimers; IMWM – intermediate molecular weight multimers; N/A – not applicable

†Novel disease-associated variants.

## Discussion

The present study revealed significant genetic heterogeneity in Croatian adult patients diagnosed with VWD. One third of identified disease-associated variants were classified as novel. In addition, simultaneous sequencing of VWF and FVIII genes provided unambiguous differentiation between VWD and mild HA.

Genetic heterogeneity was especially evident in patients classified as type-1 VWD, with disease-associated variants distributed throughout the whole VWF gene, which resulted in highly variable phenotypic laboratory results. In half of these patients, a disease-associated variant was identified despite normal levels of VWF:GPIbM, VWF:Ag, and VWF:CBA. Three patients had VWF:GPIbM levels even above 60%, which corresponds to values in healthy individuals. VWF is an acute-phase protein and its deficiency in mild forms of VWD presenting with borderline VWF levels can be masked due to the effects of age, stress, infection, or physical activity. This makes proper diagnosis based only on coagulation testing very challenging (20). Since the three patients in question were all aged above 50, normal VWF:GPIbM might be related to physiological age-related increase in plasma VWF (7). This finding shows that patients with clear clinical evidence of bleeding symptoms should still be suspected of having type-1 VWD despite normal phenotypic laboratory findings. The majority of identified causative variants in type-1 VWD were missense substitutions with an autosomally dominant inheritance. This is consistent with findings from other countries (14-17,21). Among two patients with significantly reduced levels of VWF:GPIbM, VWF:Ag, and VWF:CBA, one was as a carrier of the Vicenza variant (c.3614G>A, p.Arg1205His), which accelerates clearance of VWF and is associated with type 1C VWD. Although ultralarge multimers can remain in plasma due to variations in the VWF clearance receptor, a normal multimeric profile was found in this case (22). The severe phenotypic presentation of the other patient can be explained by the presence of a heterozygous novel variant (c.1496A>C, p.Gln499Pro) within exon 13, which affects the D2 domain of VWF. This domain is the part of the VWF propeptide responsible for VWF multimerization, intracellular transport, and secretion (23). However, even in this case the multimers were normally distributed, featuring markedly decreased intensity but without visible loss of HMWM or disproportion in VWF:GPIbM and VWF:Ag. This made the diagnosis of type-1 VWD more probable than of a qualitative subtype. The other two patients with genetic variants affecting the D2 domain exhibited a much milder laboratory phenotype. Other genetic variants resulting in a phenotype compatible with type-1 VWD affected VWF domains responsible for either binding of FVIII or GPIb, or VWF cleavage and multimerization, which confirms the complexity of the underlying causes in type-1 VWD. Besides the coding regions, the genetic basis of VWD in two patients seems to be due to disease-associated variants located within introns, hence causing disruption of normal splicing.

Type-2 VWD is genetically well-characterized, with over 200 disease-associated variants listed in the international database (4). In the majority of our type-2 VWD cases, genetic analysis confirmed the diagnosis evident from extensive phenotypic laboratory evaluation, providing the exact genetic cause. The distribution of variants associated with type-2 VWD in our study corroborated the findings from previous genetic studies (24) and showed clustering of causative variants within exon 28 for all but one patient. This patient was identified as a compound heterozygote for missense variants within exons 17 and 20. The co-presence of two variants (c.2278C>T and c.2561G>A) that reduce the binding capacity of VWF to FVIII implied the diagnosis of type-2N VWD (4). Since phenotypic laboratory results showed borderline-normal VWF and FVIII levels, as well as normal multimer distribution, as commonly seen in type-1 VWD, genetic analysis was crucial for accurate differential diagnosis. Similarly, in a patient with normal coagulation testing results and multimeric pattern, identification of the variant c.3797C>T (p.Pro1266Leu) coinherited with c.3835G>A (p.Val1279Ile) unequivocally revealed the rare subtype 2B Malmö/New York rather than type-1 VWD. The latter two variants are linked on the same haplotype and are the result of gene conversion between the VWF gene and its partial pseudogene located on chromosome 22. The patient's phenotype was mild, and the laboratory results' pattern was not associated with classic type-2B VWD and cannot be distinguished from type-1 VWD based on coagulation testing results only (25-27). Type 2A was assigned to another patient who presented with compound heterozygosity for two missense variants (c.4027A>G and c.4133C>T) affecting the A1 domain responsible for GPIb binding, thus resulting in a reduced VWF:GPIbM/VWF:Ag ratio. However, a normal multimeric pattern was observed, which implies a preserved HMWM secretion. This indicates that another cause of defective intracellular or extracellular processing is responsible for decreased VWF:GPIbM (28).

Patients with type-3 VWD, as expected, were either homozygous for null variants or heterozygous for missense or stop variants, among which three were not previously reported. The variant c.2435delC detected in two unrelated patients is the most common deletion in the VWF variant database (4), with a high prevalence in many central/northern European countries, including Sweden, Germany, Poland, and Hungary (29). We showed its presence also in patients from the Croatian geographic area.

The simultaneous analysis of VWF and FVIII genes helped us reclassify three female patients as carriers of mild HA. One of them had an additional variant within the VWF gene. As reported by Boylan et al (11), such co-presence of variants within VWF and FVIII genes may additionally complicate the patient's bleeding phenotype and interpretation of laboratory results, making the final diagnosis even more challenging.

As expected, about one third of patients with mild to normal VWF levels remained with no identified cause of their disease. This finding is in line with only about 65% of patients with VWD having a known underlying genetic variant causative of their bleeding phenotype, the likelihood of which is greater with more severe VWF deficiency (1,7). While four patients without an identified underlying genetic variant and VWF:GPIbM levels between 30% and 50% should be classified as “low VWF” (7), one patient with severely reduced both VWF and FVIII levels required further diagnostic evaluation. In order to detect possible large deletions or duplications causative of such severe phenotype, targeted multiplex ligation-dependent probe amplification analysis of the VWF gene should be performed (17), which is a further step to be undertaken and a limitation of the present report. Normal results of coagulation testing and no genetic variants found in the ten remaining patients should be a warning that the initially applied criteria for VWD diagnosis might not be accurate enough, especially when based only on mild bleeding symptoms. To avoid overdiagnosis, recent guidelines recommend that diagnosis of type-1 VWD should include strong evidence of excessive bleeding manifestations, and be inevitably coupled with VWF levels between 30% and 60% and a positive family history of VWD (20).

Another limitation pertains to the relatively small size of the studied cohort. However, patient recruitment was performed through the Reference Center for Inherited and Acquired Disorders of Hemostasis, where a vast majority of Croatian patients with bleeding disorders are monitored and treated. Furthermore, some specific laboratory tests that could have further elucidated the effect of some genetic variants, including ristocetin-induced platelet aggregation and assay that assesses the ability of VWF to bind FVIII, were not performed within the scope of this study.

In conclusion, this study revealed considerable genetic heterogeneity in adult Croatian patients diagnosed with VWD and expanded the spectrum of known disease-associated variants within the VWF gene with eleven novel ones. It also confirmed that contemporary laboratory methods still leave a proportion of patients without a defined cause of their disorder. The application of NGS of whole VWF and FVIII genes was proven as a valid approach for differential diagnosis of VWD subtypes, as well as for distinction of VWD from mild HA and identification of complex genotypes. Identification of disease-associated variants enhanced the understanding of the underlying patient disorder, allowing appropriate patient treatment and provided basis for genetic counselling.
